# Experimental and Numerical Study of Stirrup Fatigue

**DOI:** 10.3390/ma19081603

**Published:** 2026-04-16

**Authors:** Abdelwaheb Zeidi, Khaled Elleuch, Şaban Hakan Atapek, Jaroslaw Konieczny, Krzysztof Labisz, Janusz Ćwiek

**Affiliations:** 1 Laboratoire de Génie des Matériaux et Environnement, Ecole Nationale d’Ingénieurs de Sfax, Sfax University, Route Soukra Km 3.5 B.P., Sfax 1173-3038, Tunisia; 2 Laboratory of High-Temperature Materials, Department of Metallurgical and Materials Engineering, Faculty of Engineering, Kocaeli University, İzmit 41001, Türkiye; hatapek@kocaeli.edu.tr; 3 Department of Railway Transport, Faculty of Transport and Aviation Engineering, Silesian University of Technology, 40-019 Katowice, Poland; krzysztof.labisz@polsl.pl (K.L.);

**Keywords:** S235JR, punching, folding, crack growth, failure

## Abstract

Fatigue failure in scaffolding components poses significant risks to worker safety, particularly in high-altitude construction environments. This study investigates the fatigue behavior of scaffolding stirrups, a critical structural element prone to premature failure. The objective is to analyze the fatigue damage mechanisms in stirrups through a combined experimental and numerical approach. Mechanical characterization and micro-hardness testing were conducted to assess the material properties of the stirrup, while finite element modeling (FEM) was employed to simulate its performance under cyclic loading. The Johnson–Cook material model was utilized to compare experimental hysteresis curves with FEM results, validating the numerical approach. Additionally, the Extended Finite Element Method (XFEM) was applied to model crack initiation and propagation. Results reveal that material hardening and fatigue crack growth are the primary causes of stirrup failure, with distinct fatigue zones and crack paths identified. The study quantifies the relationship between crack growth stages and stirrup bending, providing insights into the failure process. These findings contribute to improving the safety and lifespan of scaffolding systems by identifying key factors influencing stirrup durability.

## 1. Introduction

Scaffolding is a temporary structural system used on the exterior of buildings, composed of wooden planks and metal poles, designed to provide workers with safe access to elevated or hard-to-reach areas during construction, maintenance, or repair operations. Since antiquity, scaffolding has remained an essential tool in the building industry, enabling efficient and secure work at height [1]. Poorly constructed or maintained scaffolding poses significant risks, including fatal accidents and severe injuries. Scaffolding is also used in suitable forms for formwork and shoring, grandstand seating, concert stages, access/observation towers, exhibition stands, ski ramps, halfpipes and art projects. Numerous safety issues have arisen from negligence during scaffolding assembly or use, as well as fatigue failure in structural components [2]. The safety and well-being of workers fundamentally depend on robust scaffolding systems constructed with components designed to withstand substantial cyclic loading. There are various techniques used in scaffolding; however, the most reliable scaffolding systems are those that incorporate components with exceptional endurance to cyclic loading. Construction companies are increasingly requesting subcontracting and help to create scaffolding; however, workplace accidents involving scaffolding result in fatal or catastrophic outcomes in over half of all cases. This is why monitoring the structural integrity of scaffolding components under high cyclic loading is a critical measure for ensuring safety and preventing failure.

Several researchers have focused on the importance of scaffolding, worker safety, and the lifespan of the process. Indeed, Ewa Blazik et al. [3] presented the attempts at determining the reasons for hazardous incidents which threaten the safety of people working on scaffolds, as well as those in their vicinity.

Kyungki Kim et al. [4] highlighted that scaffolding is a part of the temporary facilities category in construction and must be thoroughly designed, planned, procured, and managed. They developed a rule-based system that automatically plans scaffolding systems for proactive management in Building Information Modeling (BIM). Given the importance of studying fatigue phenomena, many researchers have discussed this problem, emphasizing the role of understanding, analyzing and predicting the state of parts after a few cycles. Mendoza et al. [5] used a non-destructive damage evaluation technique for carbon-fiber-reinforced polymers (CFRPs) using the virtual fields method (VFM). The results showed that specimens with measurable EG (equilibrium gap) signals exhibited considerable interlaminar matrix delamination and flexural failure.

Wan et al. proposed an efficient numerical approach based on the Extended Finite Element Method (XFEM) to analyze fatigue crack growth, demonstrating that tissue ingrowth into scaffolds significantly influences the fatigue life of fixation plates [6].

Nirmal et al. [7] investigated the impact behavior of additively manufactured AlSi10Mg alloy and the ballistic limit of projectiles using FEM simulations (Johnson–Cook model).

Welded specimens (S460MC-S460MC joint and S235JR-S235JR joint) were subjected to fatigue tests [8]; fatigue testing revealed that failure consistently occurred in the base material at the highest applied stress (625 MPa) due to localized stress concentrations, whereas the joints demonstrated superior fatigue strength at lower stress amplitudes (300 MPa), where crack initiation was delayed. This behavior aligns with conventional Wöhler curve trends, where fatigue life decreases with increasing stress amplitude. José et al. [9] conducted a two-phase fatigue performance analysis approach based on local strain and Paris’ law. Their study demonstrated that analyzing both the fatigue crack initiation and propagation phases, when combined with numerical simulations, provides a robust and suitable method for predicting the fatigue life of structural components.

While traditional approaches, such as the energy-based crack growth model proposed by Grzegorz Lesiuk et al. and Bang et al. [10,11], have laid foundational groundwork for understanding fatigue behavior, recent advancements have further refined methodologies for evaluating the fatigue performance of steel materials. For instance, studies such as those by Zhang [12] have introduced innovative numerical and experimental frameworks to assess crack propagation under cyclic loading, emphasizing the role of material microstructure and residual stresses. Similarly, research by Gbagba [13] has explored the influence of geometric discontinuities on fatigue life, providing critical insights into failure mechanisms in thin-walled structures.

Similarly, Xiaojing Li et al. [14] demonstrated the effectiveness of combining experimental analysis and numerical simulations to characterize crack growth and failure mechanisms, an approach we adopt in this study to investigate fatigue behavior in scaffolding stirrups.

SOPEM (a precision tooling and mechanical equipment company), which seeks to solve a specific problem encountered in the use of a key component in scaffolding, is one of the companies operating in scaffolding techniques. Indeed, premature damage to the stirrups during their use in scaffolding systems has occurred, causing significant concern among the company’s clients. The SOPEM company collaborates with ALTRAD for the manufacture of a product used in scaffolding. This product, known as a keyway collar (or stirrup), is intended to be welded to the railing and is installed in four parts on each floor. ALTRAD claimed there is a recurring problem affecting collars after use. Despite being made from the same materials, casting batch, and manufacturing process—and subjected to identical usage conditions—some critical scaffolding components exhibited cracking and rupture after their service period, while others remained structurally intact. The present study aims to provide a detailed analysis of stirrup fatigue, with the primary objective of identifying the root causes of failure and extending service life. This paper presents a comparative investigation, combining experimental and numerical results, to characterize crack growth behavior.

In this context, the present work distinguishes itself by combining experimental fatigue testing with advanced numerical simulations to systematically analyze stirrup failure under real-world loading conditions. Unlike prior studies that often focus on isolated aspects of fatigue, our approach integrates multi-scale stress analysis, crack growth modeling, and material characterization to propose a holistic methodology for predicting and extending the service life of scaffolding components. This not only addresses gaps in the existing literature but also offers practical recommendations for improving structural safety in high-risk applications.

## 2. Materials and Methods

### 2.1. General Context and Problematic

Scaffolding is a modular support system assembled using linear elements interconnected via keyway mechanisms, comprising superposable uprights, connecting and stiffening components, and accessories such as work platforms, consoles, and guardrails. Typically constructed from aluminum for its lightweight and durable properties, scaffolding enables efficient assembly, repositioning, and large-scale elevated work in construction, maintenance, and renovation projects.

The manufacturing process of scaffolding stirrups plays a critical role in determining their fatigue performance. Each step—such as material selection, cutting, forming, and welding—introduces residual stresses, microstructural changes, or geometric imperfections that can act as potential sites for crack initiation under cyclic loading. For instance, the forming process induces cold work hardening, which alters the local material properties and can influence fatigue life. Welding, in particular, creates heat-affected zones (HAZs) with varying microstructures and mechanical properties, often leading to reduced fatigue strength. By understanding these manufacturing-induced characteristics, the study aims to correlate process parameters with fatigue behavior, ultimately providing insights into optimizing stirrup durability and safety.

**Figure 1:**
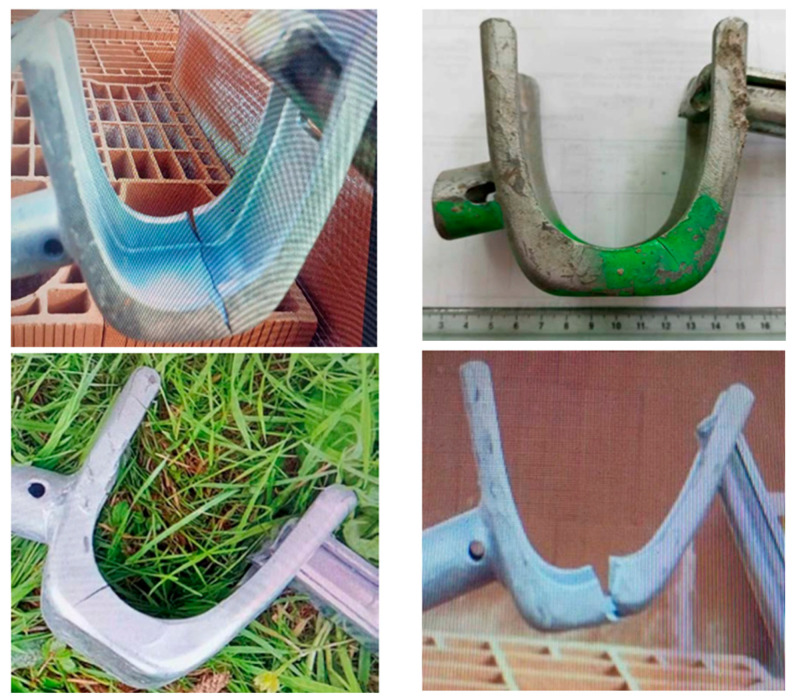
Stirrup with damage signs.

Professionals must strictly adhere to applicable safety standards to ensure structural integrity and worker protection. Fixed scaffolding is particularly adaptable to irregular surfaces, thanks to adjustable feet, and is available at various heights, with integrated guardrails to prevent falls. A critical component, the stirrup, is used in systems like the VITO 49 to securely attach guardrails to vertical pillars; it engages fully with a light tap on the key, locking the collar in place and preventing unintended movement. However, prolonged use or stress can lead to damage signs, such as cracks along the stirrup edges that propagate through the material, compromising its safety. To mitigate risks associated with fatigue failure, non-destructive testing methods, including ultrasonic testing and magnetoscopy, were utilized to detect early-stage crack initiation in scaffolding stirrups. These proactive inspections enable timely intervention and component replacement, ensuring safety before macroscopic damage becomes visible (as illustrated in Figure 1).

In real-world scaffolding applications, stirrups are subjected to complex and variable loading conditions that arise from operational activities, environmental exposure, and structural interactions. The cyclic load amplitudes experienced in service typically stem from dynamic forces such as worker movement, equipment placement, wind loads, and vibrational stresses, which can induce localized stress concentrations at critical regions like welds or geometric transitions. While the exact load magnitudes vary depending on the specific application, the chosen experimental loading scheme in this study was designed to approximate the upper range of in-service stresses, thereby accelerating fatigue damage to facilitate controlled laboratory analysis. The frequency of cyclic loading in practical scenarios is inherently linked to the usage patterns and crack initiation thresholds, as repeated loading cycles progressively degrade material integrity. Additionally, environmental conditions—such as humidity, temperature fluctuations, and corrosive exposure—can further exacerbate fatigue damage by promoting stress corrosion cracking and reducing material ductility. Though this study focuses on isolated mechanical fatigue under controlled conditions, future work will incorporate these real-world variables to provide a more comprehensive assessment of stirrup performance in service. This approach ensures that our findings remain relevant to practical applications while laying the groundwork for further investigations into long-term durability and safety optimization.

This attachment mechanism is crucial for maintaining the structural stability of scaffolding, especially under dynamic loads—such as worker movement, heavy material transport, or environmental stresses like wind and vibration. The cleat collar, as a pivotal component, must be engineered to resist a range of mechanical stresses, including shear forces, which act parallel to the surface and can cause slippage or displacement, as well as torsional forces, which induce twisting motions that could compromise the integrity of the connection. To ensure a durable and reliable assembly, the collar must be engineered using high-strength materials and precision manufacturing, ensuring it maintains a secure grip even under variable and fluctuating loads. Additionally, its locking mechanism must ensure reliable engagement, prevent unintended disengagement, and guarantee the scaffold’s stability—even under the most demanding operational conditions. Regular inspections are essential to detect any signs of wear, deformation, or fatigue, as these could undermine the collar’s performance and, consequently, the overall safety of the scaffolding system.

### 2.2. Manufacturing Process

The raw material used in this manufacturing process is hot-rolled S235JR steel, a non-alloy structural steel known for its excellent weldability, machinability, and mechanical properties, making it ideal for structural applications. This steel is supplied in the form of castings, with each casting containing 10 individual coils. These coils are produced to specific dimensions, ensuring consistency in thickness, width, and weight to meet the precise requirements of downstream processing.

Once delivered, these coils serve as the raw input material for subsequent cold-forming processes. Cold forming is a highly efficient manufacturing process that involves shaping the steel at room temperature, which enhances its strength, surface quality, and dimensional accuracy without altering its material properties. The coils undergo a series of processes, such as uncoiling, straightening, cutting, and forming, to produce components with tight tolerances and high structural integrity. This method excels in producing intricate geometries while preserving material consistency and reducing waste. The resulting cold-formed steel components are widely used in construction, automotive, and industrial applications due to their high strength-to-weight ratio and durability. It is important to note that the stirrups used in this study were designed, fabricated, and prepared by the authors, ensuring full control over the production process. This enabled a thorough understanding of potential imperfections, stress distribution, and critical features that directly impact the fatigue performance and structural integrity of the scaffolding components.

#### 2.2.1. Breakdown (Cutting)

**Figure 2:**
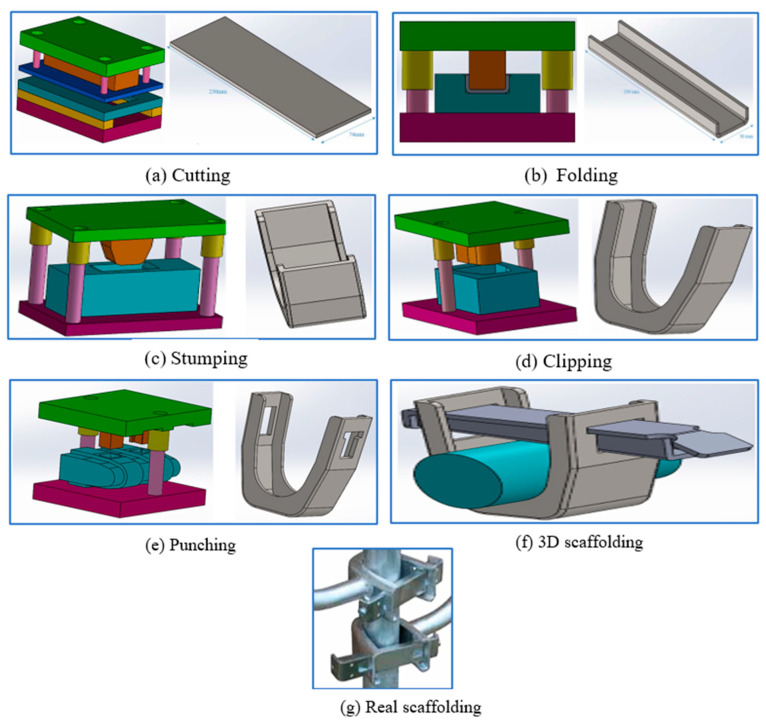
Making stirrup steps before use.

The shearing process depicted in Figure 2a involves the precise cutting of sheet metal through a mechanical action, where the material is securely clamped between a punch and a die. As the punch descends into the die, it applies a localized force that cleanly severs the material, functioning similarly to scissors but with enhanced accuracy and control. This method is essential in manufacturing, as it facilitates the production of components with smooth edges and uniform dimensional consistency.

The shearing operation demands a significant cutting force—approximately 36,966 decanewtons (daN)—due to the material’s high shear resistance. To maximize tooling efficiency and lifespan, maintaining precise punch–die clearance is essential. This gap must be meticulously adjusted to avoid problems like excessive gripping, causing tool wear or deformation, or die rupture, which can lead to costly downtime and repairs. Optimal clearance not only protects the tooling from premature wear but also ensures the production of high-quality finished components. It ensures clean, burr-free edges and dimensional accuracy, both of which are essential for downstream processes such as assembly or further machining. Additionally, maintaining the correct clearance reduces material distortion and minimizes stress on the tooling, ultimately contributing to the precision, repeatability, and overall efficiency of the shearing operation. Regular inspection and adjustment of the clearance are therefore essential to sustaining both product quality and operational reliability.

#### 2.2.2. Bending U-Shape

Folding, as illustrated in Figure 2b, produces developable parts with straight, linear folds. In this study, the required U-fold force is approximately 42,658 N. Press U bending refers to a process in which the sheet is constantly bent over the bending press twice or more to get a “U” shape. This process is accomplished by precisely controlling closing degrees of upper and lower dies and strokes to achieve the design’s exact dimensional and angular specifications. U-bending is one of the most common forming techniques in metal manufacturing industries. The process not only reduces material costs and improves production efficiency but also ensures good mechanical properties and appearance quality of products. U-bending technology is constantly updated with press evolution brake technology, which ranges from simple straight-line folding to three-dimensional folding. It not only improves the apparent bending angle and accuracy but also achieves automatic constant folding in several steps.

#### 2.2.3. Stumping

The stamping process depicted in Figure 2c is a cold plastic forming method applied to metal sheets, where the stirrup’s complex geometry—characterized by sharp angles, curves, and preformed folded sections—presents considerable challenges compared to simpler, flat shapes. In the stamping process, the pre-deformed folded sections retain residual stress from earlier plastic deformation. When subjected to additional pressure, these stresses can cause unpredictable distortions or cracking—particularly in high-stress regions where the material’s structural integrity is most compromised. To mitigate these risks, the process demands precision-engineered dies and specialized tooling, meticulously designed to conform to the folded shape while minimizing defects such as cracks or tears that arise from stress concentration. Unlike flat geometries, which feature uniform stress distribution and straightforward tooling needs, folded shapes create asymmetrical stress patterns. This increases the risk of structural vulnerabilities and demands rigorous quality control throughout the process. Non-uniform stress distribution in folded designs not only complicates the stamping process but also heightens the risk of postforming defects, such as crack propagation, which can compromise the part’s long-term performance. Thus, while flat shapes benefit from simpler tooling and more predictable outcomes, stamping intricate geometries like the stirrup necessitates advanced stress analysis, tailored tooling, and precise process control to achieve a defect-free and structurally sound final product.

#### 2.2.4. Clipping

Figure 2d highlights the clipping process, a meticulous finishing operation that trims away excess material from the edges of stamped or formed components. This step is vital for refining the part’s shape to precise dimensions and geometric accuracy, ensuring it aligns perfectly with design specifications and functional needs.

In industries like sheet metal fabrication, clipping plays a crucial role in addressing the aftermath of processes such as deep drawing, bending, or stamping. These operations often leave behind uneven edges, burrs, or surplus material—commonly referred to as ‘flash’ or ‘coast return’—due to the natural flow and deformation of the material. By systematically removing these imperfections, clipping guarantees that the final product adheres to strict tolerances and maintains the high quality standards required for its application.

Clipping is a critical finishing process that refines rough or irregular edges, transforming them into precise, clean profiles. By removing excess material, it ensures dimensional accuracy, superior surface quality, and optimal fit—preparing the part for seamless assembly or immediate use in final applications.

Whether integrated as an in-die cutting step during stamping or executed as a secondary operation, clipping is essential for converting raw stamped blanks into high-precision components that meet rigorous engineering standards. Failure to perform this step could leave residual excess material, potentially compromising functionality, assembly compatibility, or aesthetic appeal—especially in high-stakes industries like automotive, aerospace, and construction, where precision and reliability are non-negotiable.

The quality of clipping directly influences the performance, safety, and durability of the final product, reinforcing its indispensable role in advanced metalworking.

#### 2.2.5. Punching

Punching, depicted as the final manufacturing operation in Figure 2e, is a high-precision process used to add intricate details—such as holes, slots, or custom cuts—to preformed parts with exceptional accuracy. This process utilizes a hardened punch to forcibly shear a precise section of material from a sheet metal workpiece or strip, with a supporting die ensuring a clean, controlled separation. Punching is renowned for its speed, efficiency, and cost-effectiveness, making it a preferred method for producing high-quality features without the need for expensive tooling or secondary operations. The simplicity and affordability of punching tools further enhance their appeal, offering manufacturers a budget-friendly solution for achieving precise, repeatable results in mass production.

In scaffolding applications, punching is used to form functional recesses—such as the two precision-cut openings in the stirrup—facilitating smooth assembly and compatibility with adjacent scaffolding components. Following these forming processes, components may undergo galvanization to enhance corrosion resistance, while others are dispatched in their raw state, depending on their intended application and environmental exposure.

Figure 2f highlights additional critical scaffolding components: a key and a 48.8 mm diameter cylinder, both essential for structural stability and operational safety. However, despite their robust design, these components—particularly the stirrup (collet)—are subjected to intense mechanical stresses during use, as illustrated in Figure 2g. Over time, repeated cyclic loading can induce fatigue, leading to the formation of micro-cracks that propagate under operational strains. These cracks not only compromise the structural integrity of the stirrup but also pose significant safety risks, particularly in high-altitude work environments where scaffolding failures can result in catastrophic accidents.

The emergence of cracks necessitates immediate intervention, often causing unplanned work stoppages and costly downtime for repairs or replacements. Fatigue-induced degradation underscores the importance of regular inspections, preventive maintenance, and material quality control to mitigate risks and ensure the longevity and reliability of scaffolding systems. Ultimately, the safety of workers operating at height depends on the durability and resilience of these components, making fatigue resistance and structural monitoring critical priorities in scaffolding design and maintenance.

The manufacturing process of S235JR steel stirrups plays a pivotal role in their fatigue performance, primarily due to the introduction of residual stresses and geometric notches that act as preferential sites for crack initiation. During forming and welding, non-uniform cooling and plastic deformation generate tensile residual stresses near weld zones and sharp transitions, significantly reducing the material’s fatigue resistance by lowering the effective stress threshold for crack nucleation. Additionally, notch effects—arising from abrupt changes in geometry, such as at fillet radii or weld toes—create localized stress concentrations that further exacerbate fatigue damage by amplifying applied cyclic loads. These manufacturing-induced imperfections are critical, as they accelerate the transition from micro-crack initiation to macroscopic propagation, ultimately compromising the structural integrity of the stirrup under service conditions. This discussion underscores the necessity of considering manufacturing processes as a key variable in fatigue analysis, bridging the gap between production methods and in-service performance.

### 2.3. Material Characterization

The fatigue behavior of S235JR steel stirrups in this study is examined through a comprehensive analysis that integrates manufacturing influences, material characterization, and advanced numerical modeling. The manufacturing process, including forming and welding, introduces geometric discontinuities and residual stresses that serve as preferential sites for crack initiation, thereby directly impacting fatigue life. To ensure clarity and relevance, the discussion now focuses on these critical aspects, omitting less pertinent details. Material characterization was conducted in accordance with ISO 6892-1, (Paris, December 2019) with reported mechanical properties reflecting batch-specific variations due to processing history, such as cold-working effects. Any deviations from standard S235JR values are justified based on these factors, and the experimental procedures—including sample preparation, testing conditions, and statistical analysis—are thoroughly documented to ensure reproducibility.

The numerical model employs the Johnson–Cook failure criterion within an XFEM framework, with boundary conditions replicating the experimental setup: fixed constraints at the grips and cyclic loading applied at the stirrup’s mid-span. A refined mesh was used in critical regions to capture stress gradients accurately, while coarser elements optimized computational efficiency. Model parameters were calibrated using an inverse method, validated by comparing simulated crack propagation and load–displacement curves with experimental data. Damage evolution was tracked using STATUSXFEM, PHILSM, and PSILSM variables, providing a robust description of crack geometry and progression. This integrated approach establishes a clear connection between manufacturing, material behavior, and numerical predictions, laying the foundation for future refinements that will incorporate microstructural and environmental influences to further enhance the model’s accuracy and applicability.

The stirrup under examination is constructed from S235JR steel, a low-carbon, non-alloy structural steel renowned for its versatility, weldability, and mechanical strength, with its chemical composition precisely outlined in Table 1 and verified by the authors through stationary SPECTROMAXx spectroscopic metal analysis (Spectro, Germany) to ensure elemental accuracy. As a material compliant with EN 10025-2 standards, S235JR is extensively employed across a wide range of industries, including industrial piping and pipelines for gas and oil transport; marine applications such as shipbuilding, agricultural machinery requiring durability; construction equipment like beams and scaffolding; and pressure vessel manufacturing, where its ability to withstand internal pressures is critical [15]. The steel’s performance is significantly enhanced by its chemical composition, particularly the presence of copper (Cu) and manganese (Mn); copper improves corrosion resistance and longevity in challenging environments, while manganese boosts hardness, tensile strength, and wear resistance, ensuring the material can endure mechanical stresses and cyclic loading without premature failure. This balanced combination of properties—coupled with its cost-effectiveness, machinability, and formability—makes S235JR an optimal choice for structural components like the stirrup, where safety, precision, and long-term reliability are essential to maintaining operational integrity in demanding applications.

**Figure 3:**
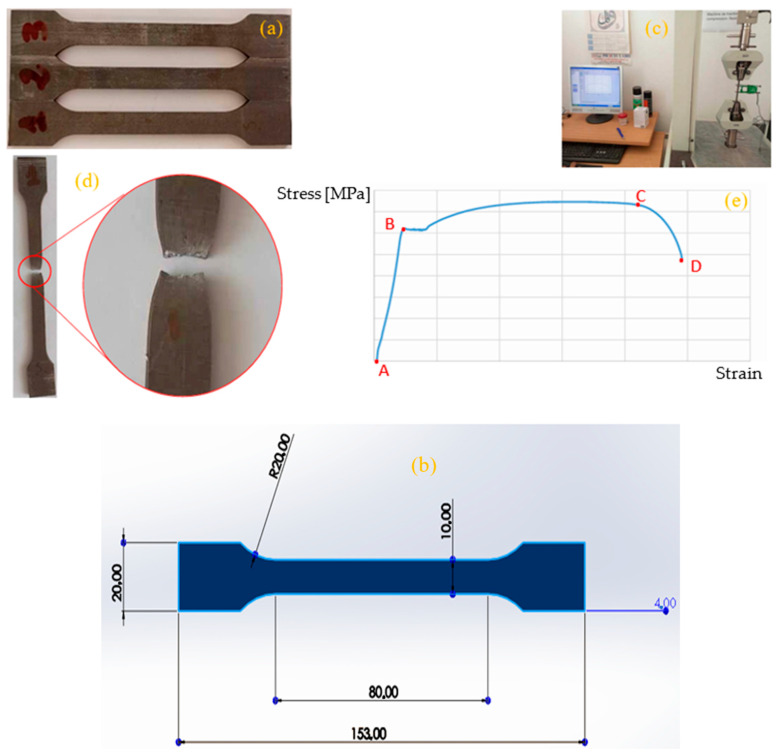
Experimental setup and tensile test results: (**a**) Specimens, (**b**) specimen dimensions, (**c**) testing machine, (**d**) specimen after tensile test, (**e**) stress–strain curve.

Understanding the mechanical properties of materials, such as S235JR steel, is critical for both engineering design and numerical simulations, as these properties define how a material behaves under stress, including its stiffness, elongation, toughness, and resistance to mechanical loads, all of which are essential for predicting performance in real-world applications. To obtain these properties, three standardized test samples—shown in Figure 3a—were prepared, with their dimensions, including the active gauge length, detailed in Figure 3b, to ensure consistency with prior results. The test samples used in this study were prepared by cutting sheet metal made of S235JR steel, rather than being extracted from pre-existing stirrups, to ensure uniformity in material properties and dimensional accuracy. The cyclic loading amplitude of ±5000 N was selected to simulate upper-bound service conditions typical of scaffolding stirrups, where dynamic operational loads—such as those induced by worker activity, equipment placement, and vibrational stresses—can generate localized stress concentrations conducive to fatigue failure. This load level was determined based on preliminary finite element analyses and field observations, ensuring that the experimental conditions remain representative of real-world stress magnitudes while accelerating damage progression for a controlled laboratory study. The displacement rates of 100 mm/min and 200 mm/min were chosen to evaluate strain rate sensitivity in the material’s fatigue response, with the lower rate approximating quasi-static loading and the higher rate reflecting more dynamic operational conditions. These rates also facilitated practical testing constraints while ensuring that the observed crack initiation and propagation behaviors remained within the validity range of the Johnson–Cook model, which accounts for strain rate effects. Future work will further refine these parameters to incorporate variable loading profiles and environmental influences, enhancing the applicability of the findings to in-service conditions. Testing was conducted using a tensile machine (Figure 3c), where each sample was subjected to a controlled tensile force until fracture (Figure 3d) under room temperature conditions (298 K) and a constant loading velocity to maintain accuracy. The resulting stress–strain curve (Figure 3e) reveals three key phases: the elastic deformation zone (AB), where the material deforms reversibly; the plastic deformation zone (BC), where permanent deformation occurs; and the rupture zone (CD), where the material ultimately fails after reaching its maximum resistance. This curve confirms that S235JR steel exhibits an average yield strength of approximately 457 MPa, aligning with its classification as a low-carbon structural steel. The primary goal of these mechanical tests is to quantify material properties—summarized in Table 2—which are then used in strength calculations and Finite Element Method (FEM) simulations to ensure structural reliability and performance optimization in engineering applications.

The Johnson–Cook (JC) model, introduced by Johnson and Cook in 1983 [16], is an empirical constitutive model widely used to describe the stress–strain relationship and thermo-viscoplastic behavior of materials, including S235JR steel. Its simplicity, robustness, and ease of implementation make it particularly suitable for simulating material behavior under dynamic loading conditions, such as high strain rates and elevated temperatures.

In this study, the JC model is employed to analyze the fatigue and failure mechanisms of scaffolding stirrups, where dynamic loading and stress concentration play critical roles. The model’s ability to incorporate strain rate and temperature effects ensures reliable predictions of material response, supporting the optimization of structural design and safety in scaffolding systems. The mathematical formulation of the Johnson–Cook model, as presented in Equation (1), expresses the flow stress as a function of strain, strain rate, and temperature

(1) $$\sigma =(A+B\varepsilon^{n})(1+C\ln\frac{\dot{\varepsilon }}{{\dot{\varepsilon }}_{r}}){\left(1-\frac{T-T_{r}}{T_{m}-T_{r}}\right)}^{m}$$

where A is the yield stress, B is a material constant, n is the hardening coefficient, C is the stress–strain sensitivity, m is the temperature coefficient, $\dot{\varepsilon_{0}}$ is the reference strain rate and $\dot{\varepsilon_{p}}$ is the plastic strain rate. T is the current temperature; T_r_ is a reference temperature and T_m_ is a reference melt temperature. Johnson–Cook model parameters are defined in Table 2.

In Equation (1) and from left to right, the first term characterizes the elastoplastic behavior of Ludwick’s law (the strain hardening effect). The second term considers the viscoplasticity (strain rate strengthening) and finally, the third term quantifies the temperature effect on the behavior of the material.

In terms of the experimental conditions, the reference strain rate, $\dot{\varepsilon }$_r_, and the reference temperature, T_r_, were taken as 1.0 s^−1^ and 1298 K, respectively. Johnson and Cook agreed that fracture strain mainly depends on the stress triaxiality ratio, strain rate and temperature.

The Johnson–Cook (JC) model parameters were calibrated using an inverse method, where experimental stress–strain data were employed to iteratively adjust the model’s constitutive parameters. This process involved optimizing the yield strength (A), strain hardening coefficients (B, n), strain rate sensitivity (C), and thermal-softening exponent (m) to achieve the best fit between simulated and experimental results. The calibration was validated by ensuring that the numerical model accurately replicated key mechanical responses, including stress–strain behavior, crack initiation thresholds, and failure progression observed in the fatigue tests. This systematic approach guaranteed that the JC model reliably represents the dynamic and failure characteristics of S235JR steel under cyclic loading conditions.

The Extended Finite Element Method (XFEM) represents a powerful advancement over the traditional Finite Element Method (FEM), specifically designed to simulate complex fracture mechanics phenomena, including crack initiation, propagation, and branching in materials [17]. Within the ABAQUS simulation environment, XFEM is implemented as an Interaction Model, enabling engineers and researchers to accurately model dynamic crack behavior under various loading conditions. Unlike conventional FEM, which requires explicit mesh refinement around crack tips and frequent remeshing as cracks evolve, XFEM eliminates these computational limitations by introducing enrichment functions directly into the finite element formulation.

The Johnson–Cook (JC) model was implemented to capture the strain rate and temperature-dependent plastic behavior of the stirrup material under cyclic loading. The model parameters (A, B, n, C, and m) were identified through an inverse calibration process, using experimental stress–strain data obtained from tensile tests at varying strain rates (0.001–10 s^−1^) and temperatures (20–200 °C). The calibrated JC model was then integrated into the finite element software (Abaqus/Explicit 6.17) to simulate the hysteresis loops observed during fatigue testing, with a focus on predicting the hardening behavior and localized plastic deformation.

For crack growth analysis, the Extended Finite Element Method (XFEM) was employed to model crack initiation and propagation without remeshing. The XFEM implementation utilized a cohesive zone model to define the fracture properties. The boundary conditions in the numerical model replicated the experimental setup: the stirrup was fixed at one end, and a cyclic displacement load was applied at the opposite end to simulate in-service conditions. The mesh was refined in the expected crack propagation zones, with a minimum element size of 0.2 mm to ensure accurate stress distribution and crack path prediction. Sensitivity analyses were conducted to validate the mesh independence of the results.

The S235JR steel was selected for this study due to its prevalence in structural applications, particularly in scaffolding systems, where its combination of strength, ductility, and cost-efficiency makes it an industry standard. Its well-characterized mechanical properties, including a yield strength of 235 MPa and good fatigue resistance, provide a robust foundation for analyzing fatigue crack propagation and failure mechanisms in real-world conditions.

For the Johnson–Cook (JC) model parameters, the values were carefully chosen to reflect the material’s behavior under dynamic and high-strain-rate conditions. The yield stress (A) aligns with the nominal yield strength of S235JR, while the strain hardening coefficient (B) and exponent (n) were calibrated to capture the material’s work-hardening response during cyclic loading. The strain rate sensitivity (C) accounts for the increased strength under rapid loading, and the thermal-softening exponent (m) models strength reduction at elevated temperatures, both of which are critical for simulating real-world operational stresses. These parameters ensure that the JC model accurately represents the material’s response to complex loading scenarios, enabling reliable predictions of fatigue life and structural integrity in scaffolding components.

In the ABAQUS software, 6.17 these enrichment functions are integrated to capture discontinuities—such as cracks, voids, or material interfaces—without altering the underlying mesh structure [18]. This is achieved using specialized shape functions that incorporate discontinuous fields and asymptotic crack-tip fields, allowing for the realistic representation of crack growth along arbitrary, unpredictable paths. The key advantage of XFEM lies in its ability to simulate crack propagation independently of the mesh, meaning that cracks can initiate and extend freely through the material without requiring manual mesh adjustments or computationally expensive remeshing procedures. This feature not only reduces simulation time and complexity but also enhances the accuracy of predictions, particularly in scenarios involving complex geometries, heterogeneous materials, or dynamic loading conditions.

The XFEM model in ABAQUS was developed with careful consideration of geometric simplification, mesh design, and boundary conditions to ensure accurate simulation of crack initiation and propagation. To reduce computational cost while maintaining accuracy, an axisymmetric model was employed, simplifying the stirrup geometry to half of its full structure based on symmetry. A tetrahedral mesh was selected for its ability to conform to the complex geometry of the stirrup, particularly around critical regions such as contact surfaces, where stress concentrations are most pronounced. The mesh was generated using a top-down partitioning approach, with refined elements applied to high-stress zones to capture localized deformation and crack growth, while coarser elements were used in less critical areas to optimize computational efficiency. Surface-to-surface contact was defined between the pin and stirrup to realistically simulate load transfer and frictional interactions. For boundary conditions, the stirrup was fully fixed at its lower surface, while a cyclic force was applied to the upper surface to replicate experimental loading. This setup ensured that the model accurately represented the physical constraints and loading scenarios observed in the fatigue tests.

By leveraging XFEM, engineers can study fracture mechanics in greater detail, including stress intensity factors, crack growth rates, and failure modes, which are critical for assessing structural integrity in applications such as aerospace components, automotive safety systems, civil infrastructure, and mechanical assemblies. The method’s versatility also extends to multi-physics simulations, where thermal, mechanical, and environmental effects interact to influence crack behavior. Ultimately, XFEM provides a robust and efficient tool for predicting and mitigating material failure, enabling the development of safer, more durable, and optimized designs across a wide range of industries.

Numerical simulation offers a low-cost alternative for obtaining reliable results without the necessity of conducting physical experiments. The results of simulation are highly recognized by researchers in the industrial world. Understanding the fatigue phenomenon in the material can be achieved through crack growth study. In fact, the ABAQUS software was used, using the Finite Element Method (FEM) and under the heading ‘XFEM’. This method allows mechanical stress on the one hand and cyclic loading on the other hand. The initiation phase is the first one that occurs during crack growth. This phase begins following material fatigue under cyclic stresses and excessive loads during scaffolding. Geometric or microstructural defects, as well as weak zones created by imperfections and stress concentrations, can give rise to this crack.

**Table 2:** Mechanical properties and Johnson–Cook parameters of S235JR [19,20].

Description	Notations	Value
Young modulus	E	209 GPa
Poisson’s ratio	ν	0.3
Density	ρ	8587 Kg/m^3^
Tensile strength	R_m_	426 MPa
Elongation	A%	35
Yield stress	Re	357 MPa
Yield stress constant	A	480 MPa
Strain hardening	B	153 MPa
Constant	n	0.36
Viscous effect	C	0.0141
Thermal-softening constant	m	1.3
Reference strain rate	$\dot{\varepsilon_{0}}$	1 s^−1^
Melting temperature	T_m_	1773 K
Reference temperature	T_r_	1298 K

## 3. Results and Discussion

### 3.1. Experimental Results

**Figure 4:**
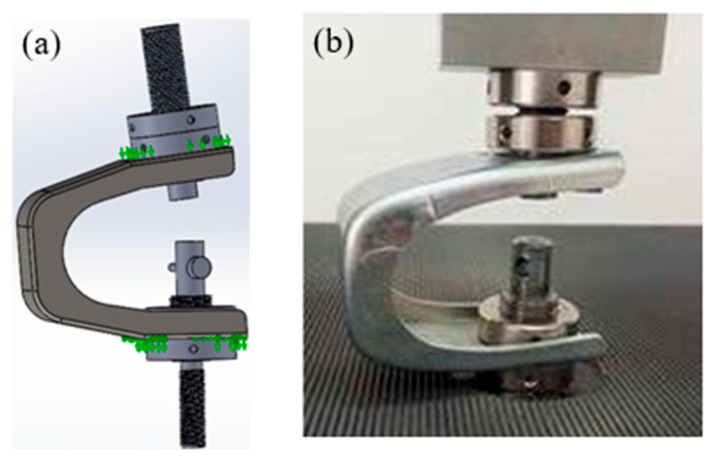
Laboratory simulation conditions for stirrup without axis: (**a**) 3D-modeled case, (**b**) real case.

Transitioning from an industrial problem to laboratory-based experimental or numerical simulation presents significant challenges. To accurately analyze and understand system defects, it is critical to replicate real-world loads and boundary conditions as closely as possible. This requires careful simplification of the problem—without overlooking key operational realities. In scaffolding systems, the stirrup is used in conjunction with a rod to connect two axes. In the laboratory, as illustrated in Figure 4, stress conditions were adapted with controlled modifications to closely mirror real-world scenarios. A tensile machine was employed to perform tensile-compression tests on the stirrup component. Selecting appropriate test parameters—such as the loading force and speed—is a pivotal step that demands precision, as exceeding optimal values could compromise the validity of the results.

In the field of metal fatigue, service life is divided into two main phases, namely crack initiation and propagation [21,22]. The tests were carried out in the laboratory using a tensile machine. This was equipped with clamping parts (threaded rod and nut) and a 45 kN load cell was used. Two cyclic compression–decompression (tension) tests were carried out, like the action of striking a key to move it and press it against the cylinder with its conical shape. Thus, the part is subjected to compression, and the cylinder in the middle, clamped by a key collar, reacts with the opposite compression (Figure 4a,b). This is like a tensile test.

**Figure 5:**
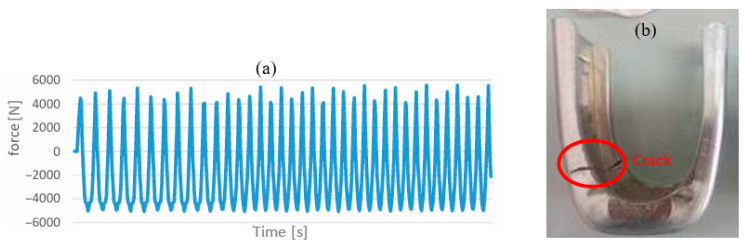
Tensile–compression test without axis using tensile machine: (**a**) force–time curve, (**b**) stirrup fatigue.

In the first step, a test without a cylinder was carried out with a strain rate of 100 mm/min, over several about 1000 cycles. Stresses applied during the test are of the sinusoidal type, by applying a load of 5000 N for limit cycle 1 and −5000 N for limit cycle 2, as shown in Figure 5a. The end of each test is marked by a crack’s appearance and its propagation. Currently, after 286 cycles, the already mentioned signs appear, which require the experiment to be closed.

Although initial crack formation was observed at 286 cycles (Figure 5a), the fatigue test was continued until the stirrup reached complete rupture after several thousand load cycles, ensuring a comprehensive analysis of its fatigue life.

**Figure 6:**
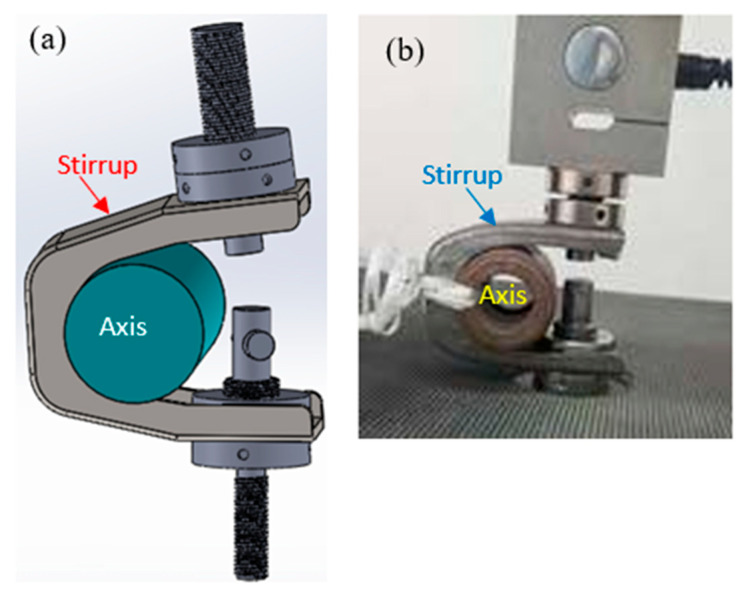
Laboratory simulation conditions for stirrup using axis: (**a**) 3D-modeled case, (**b**) real case.

For the second test, an axis with a 53 mm diameter—matching the standard dimensions used in scaffolding—was employed to replicate real-world conditions. The experiment was conducted at a deformation speed of 200 mm/min, chosen to accelerate testing while maintaining controlled conditions. Over approximately 1000 cycles (Figure 6a,b), the test aimed to simulate repetitive operational stresses, such as those caused by worker movement or environmental vibrations. This approach allowed for an efficient yet accurate assessment of the material’s fatigue behavior, crack initiation, and structural integrity under prolonged cyclic loading.

**Figure 7:**
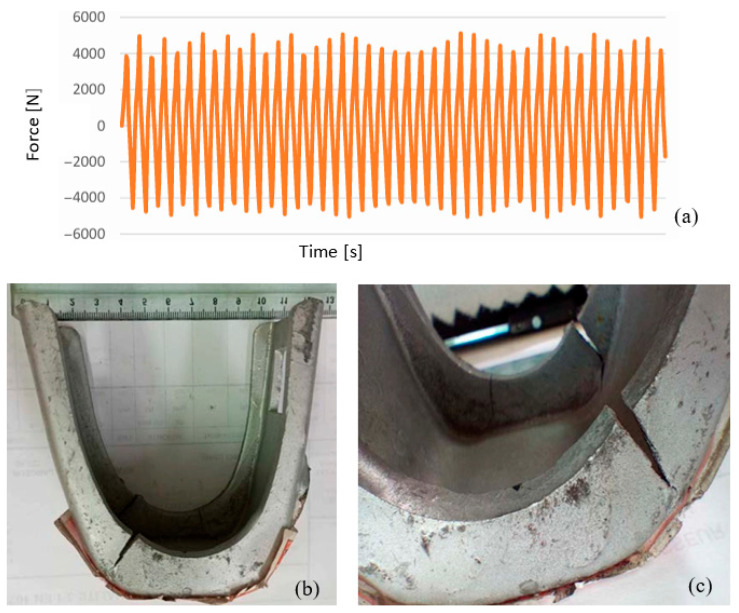
Tensile–compression test with axis using tensile machine: (**a**) Force–time curve; (**b**,**c**) stirrup fatigue (cracks).

The test was conducted by applying a load of F = 5000 N for limit cycle 1 and F = −5000 N for limit cycle 2 as shown in Figure 7a. It is noted that the part (stirrup) reached rupture after 660 cycles in the zone as illustrated in Figure 7b. From the two tests carried out in the fatigue study, it was noticed that the product (stirrup) presented damage (crack) in the same area. However, the cylinder axis allows them to increase their service life, since in the test without cylinders they support less load before plastically deforming or developing cracks, which reduces their number of cycles.

**Figure 8:**
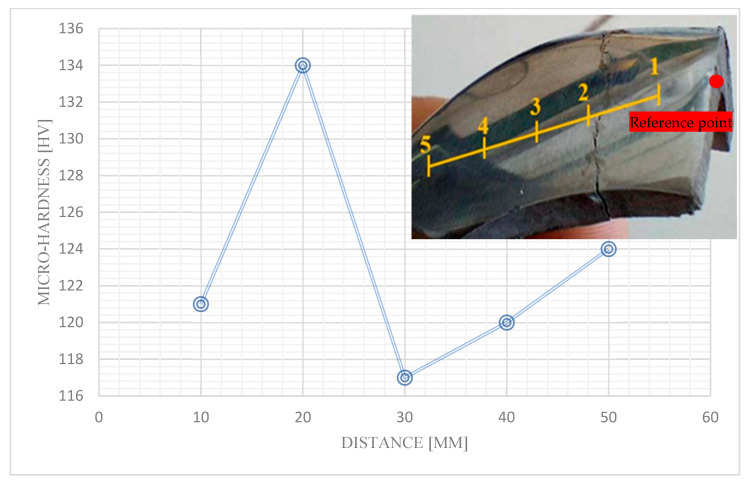
Variation in micro-hardness with distance from the reference point in the scaffolding stirrup.

Figure 8 illustrates a transverse fatigue crack propagating through the stirrup, oriented perpendicular to the principal loading direction. The crack initiates in a stress concentration region—likely due to geometric discontinuities or microstructural defects—and propagates under cyclic loading, following a Mode I (opening) fracture mechanism. Given its transverse alignment, the crack compromises the load-bearing capacity of the stirrup by reducing its effective cross-sectional area, ultimately leading to catastrophic failure through complete separation. This fracture mode renders the component structurally unsound, necessitating immediate replacement to prevent operational hazards in scaffolding systems. The observed crack morphology aligns with classical fatigue failure patterns in ductile metals, confirming the critical role of stress distribution and material defects in fatigue life prediction. Five micro-hardness tests in Hv were carried out near the crack zone. The curve (Figure 8) presents the micro-hardness measurement results. Indeed, these show a peak for the second test (~134 hv) which is the result of the test closest to the crack. Micro-hardness decreases in the low-toughness zone by gradually moving away from the macro-crack. This result can be explained by the hardening phenomenon proposed by [23,24] when studying the micro-hardness and microstructure of different steels hardened by a fiber laser. The micro-hardness measurements revealed a gradual decrease in hardness with increasing distance from the macro-crack, a phenomenon attributed to localized strain hardening induced by cyclic plastic deformation during fatigue loading. Unlike the uniform hardening achieved through fiber laser treatment, where rapid heating and quenching produce a martensitic microstructure, the observed hardening in this study results from dislocation accumulation and grain refinement at the crack tip. Under bending-fatigue conditions, repeated stress cycles concentrate plastic deformation in these regions, leading to enhanced dislocation density and localized work hardening. This process elevates the hardness near the crack but diminishes gradually away from it, reflecting the non-uniform distribution of plastic strain within the material. Such hardening behavior significantly influences crack propagation resistance and, consequently, the fatigue life of the scaffolding stirrups, underscoring the importance of microstructural evolution in fatigue failure analysis. Thus, following cyclic loading (compression–traction) exerted on the stirrup, the micro-hardness of the stress concentration area increases and subsequent hardening takes place. The fatigue-induced crack propagation observed in the stirrup follows a coalescence mechanism, where micro-cracks initiate at stress concentration sites, propagate under cyclic loading, and eventually coalesce to form a macroscopic fracture plane. This process results in the complete separation of the stirrup into two distinct segments, effectively eliminating its load-bearing capacity. Structurally, this failure mode renders the component incapable of sustaining the required scaffolding forces, compromising the system’s integrity. From an operational perspective, the necessity of replacing or reinforcing the failed component introduces logistical challenges and heightened safety risks, as the compromised stirrup may lead to unstable load distribution. These findings emphasize the critical need for proactive fatigue monitoring and material optimization to mitigate failure risks in high-stress structural applications.

### 3.2. Numerical Results

**Figure 9:**
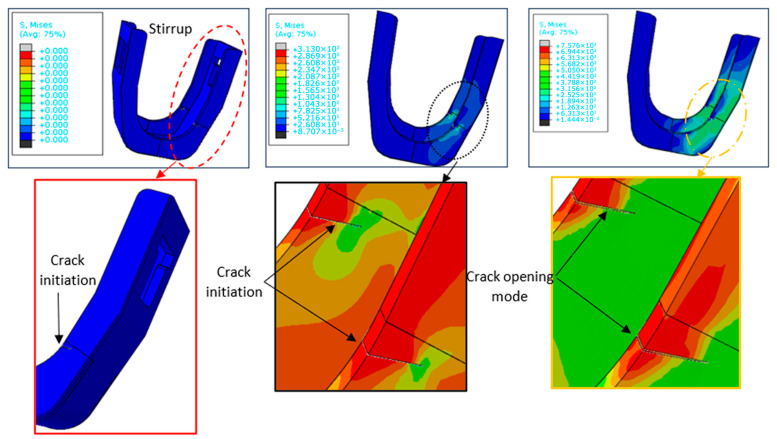
Investigation of crack initiation in scaffolding stirrups.

As shown in Figure 9, the crack occurs in the opening mode with a somewhat lengthy process since it depends mainly on internal factors such as the hardness and Young’s modulus of S235JR and other external factors such as the environment, humidity, temperature, and usage rate (number of cycles). This first phase is very difficult to detect during use, which represents an inevitable danger.

In multi-cycle fatigue scenarios, crack initiation tends to be a gradual process and is often challenging to detect during its initial phase.

**Figure 10:**
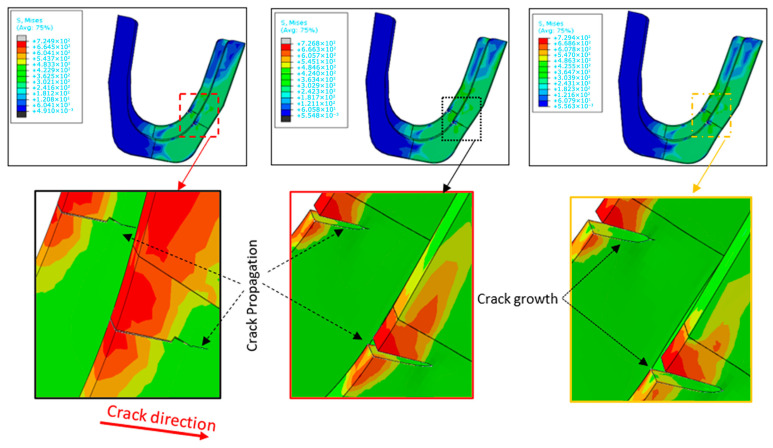
Investigation of stable crack propagation in S235JR steel stirrups: Experimental characterization and XFEM-based predictions.

Following crack initiation, the process enters the propagation phase, known as the ‘stable crack growth phase.’ In the stirrup, the crack advances incrementally with each load cycle. This phase can be further divided into two sub-stages, each characterized by distinct propagation behavior. The first phase is generally influenced by the material’s microstructure and results in short cracks [25,26]. These join to form longer cracks. The second sub-phase represents a high growth rate with longer and more stable cracks. The crack present in the stirrup is the result of in-service fatigue, as it is a material with ductile fracture. In the present phase the stable crack growth is slowly controlled; indeed, the crack propagates at a fixed rate when the load is increased. In addition, in this case, sudden failure does not occur since the material is able to absorb energy. The simulation results reveal that the S235JR steel stirrup exhibits a progressive failure mechanism rather than sudden fracture, a behavior attributed to its capacity for energy absorption through plastic deformation. This is evidenced by the gradual increase in strain energy density observed in the XFEM analysis, where the material undergoes stable crack propagation under cyclic loading. The stress–strain curves derived from the simulations demonstrate a ductile response, characterized by a prolonged plastic deformation phase prior to final separation. Additionally, the damage evolution plots indicate that energy is dissipated through microstructural mechanisms, such as dislocation movement and localized hardening, which delay catastrophic failure. These findings align with the material’s known ductility but are specifically quantified and visualized through our numerical model, confirming its ability to mitigate abrupt structural collapse. The crack length increases but it does not enter an unstable field, and it occurs without rapid acceleration. The crack size does not reach a critical value and the material’s ability to absorb energy is not exceeded. Figure 10 shows what has just been said. Currently, despite the increase in von Mises stress, which reflects the increase in cyclical loading, the crack does not lead to damage or catastrophic failure. In this stage of crack propagation, the stirrup retains its functional integrity, maintaining operator safety on the scaffolding structure. Nevertheless, vigilance is required, as the crack can quickly progress into the unstable propagation phase, posing a potential risk.

**Figure 11:**
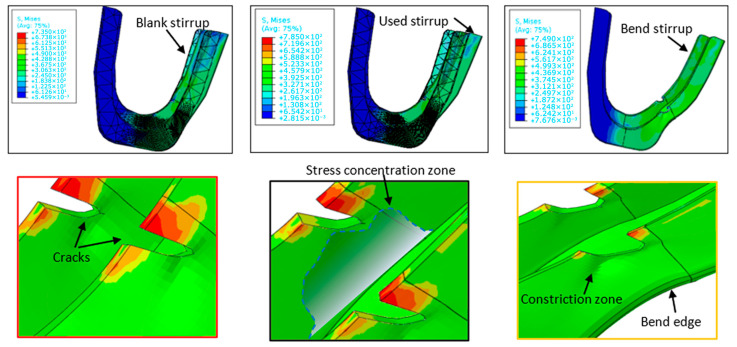
Unstable crack growth and final fracture in scaffolding stirrups: Fatigue failure dynamics under cyclic loading.

Following the stable-crack-growth phase, the crack transitions into an unstable state. If it reaches a specific threshold referred to as the ‘critical crack size’, catastrophic failure may ensue. Upon reaching the unstable-growth phase, the crack propagates rapidly through the grain boundaries of the material or by dissociating the grains themselves which was shown by Chai et al. [27] using acoustic emission and Zhang and collaborators [28] through the CJP Model (Christopher James Patterson Model). In the S235JR steel stirrups investigated in this study, crack propagation during the unstable-growth phase exhibits a mixed-mode fracture behavior, combining both intergranular and transgranular mechanisms. Preliminary observations from macroscopic examination and simulation results suggest that cracks initially follow grain boundaries in regions of high stress concentration, particularly where microstructural inhomogeneities or inclusions act as nucleation sites. As the crack advances, it transitions to transgranular propagation, cleaving through grains under the influence of localized plastic deformation and stress intensification. The resulting fracture surfaces display characteristics typical of ductile failure, including dimpled rupture zones indicative of microvoid coalescence, alongside smoother, faceted regions associated with intergranular separation [29]. This dual propagation mode reflects the material’s response to cyclic loading, where grain boundary weakness and intragranular slip systems collectively govern the final fracture morphology. Further microstructural analysis, including SEM imaging, will be presented in subsequent work to provide a more detailed characterization of these mechanisms. Material toughness in this phase is exceeded by the local stress near the crack. In the case of the S235JR stirrup, crack propagation is unpredictable both in size and direction, which inevitably results in the failure of the component. A dramatic and rapid crack acceleration can occur, generating new fracture surfaces and greatly diminishing, or entirely negating, the material’s ability to absorb energy. This type of cracking occurs with little warning, so the lives of stirrup users can be in danger. Given that human life is a paramount consideration in all engineering projects, controlling the unstable phase of crack propagation is essential to mitigate risks and ensure safety. As shown in Figure 11, from a von Mises stress of 735 MPa the folding phenomenon begins to appear on the part subjected to intense cyclic loading. At the periphery of the crack, stress concentration and geometric constriction zones develop, which play a key role in promoting crack propagation. Following stress concentration, work hardening is induced, leading to the generation of dislocations that first appear on the surface of the steel and progressively develop throughout the bulk of the stirrup. An anti-plane shear mode (mode III) seems to be occurring. Once this stage of crack propagation is reached, the stirrup can no longer reliably support the applied loads in the scaffolding system. Therefore, it is essential to avoid the transition into unstable crack growth [30].

**Figure 12:**
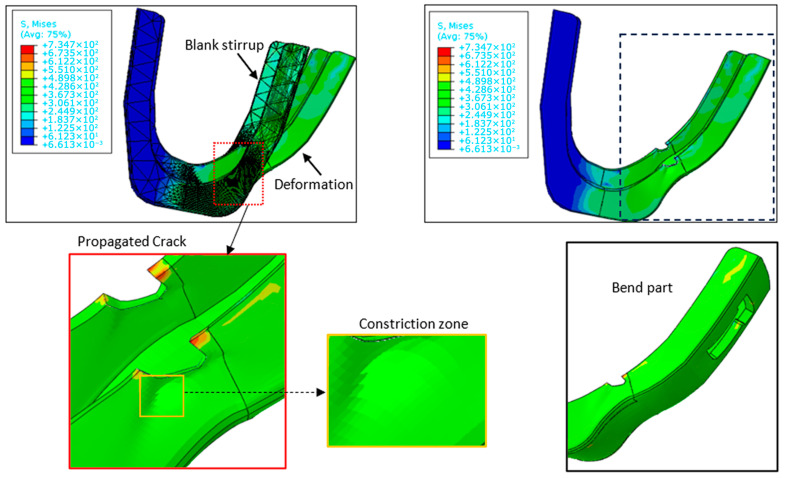
Investigation of fatigue-induced failure in S235JR steel stirrups: Focus on constriction, bending, and crack growth mechanisms.

The following section details the final rupture phase of the stirrup. Indeed, as shown in Figure 12, the loaded region undergoes bending, leading to the emergence of striations and localized stress concentrations. The increased crack size compared to its initial state has contributed to the enhanced deformation of the stirrup. Crack propagation occurs in both stable and unstable states, confirming the damage and eventual failure of the component. Consequently, the part is no longer fit for service and requires replacement. To conclude, analyzing crack growth, including initiation and propagation phases, provided valuable insights into the stirrup’s in-service behavior and a comprehensive understanding of the crack propagation mechanisms across all stages. In this study, the crack growth analysis of S235JR steel stirrups revealed distinct behaviors during the initiation and propagation phases. Crack initiation was predominantly observed at stress concentration sites, such as geometric discontinuities or material defects, where localized plastic deformation facilitated micro-crack formation. During the propagation phase, cracks exhibited a transition from stable, slow growth—governed by cyclic loading and strain hardening—to rapid, unstable propagation as the effective cross-sectional area diminished. Simulation results further confirmed that the energy absorption capacity of the material delayed sudden failure, allowing for a gradual degradation of structural integrity. While this study provides a foundational understanding of these mechanisms, a detailed microstructural analysis (including fracture surface morphology and SEM observations) will be presented in subsequent work to fully elucidate the material-specific crack propagation behavior.

**Figure 13:**
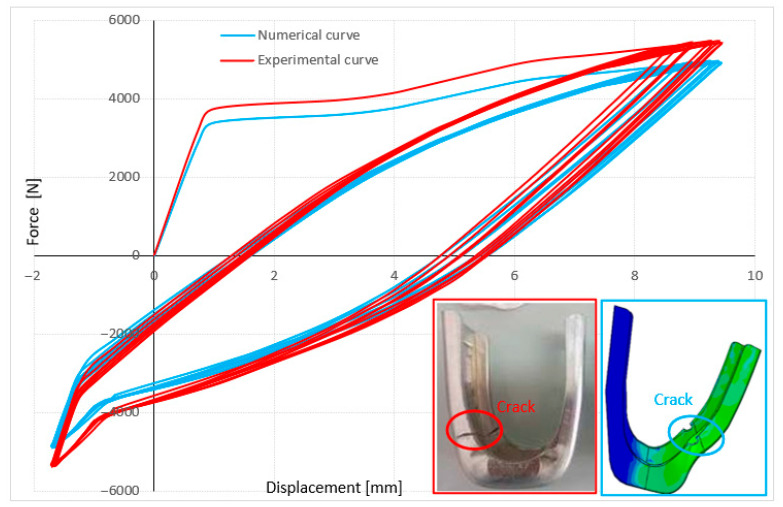
Numerical and experimental hysteresis curves under compression–tensile loading.

The stirrup was loaded under displacement control with an amplitude of 11 mm. This loading induced plastic deformation in the stirrup, with hysteresis curves reflecting the influence of stress concentration. In Figure 13, following crack initiation on the stirrup, a rapid increase in the softening zone intensity was observed, culminating in the ultimate failure of the specimen. The stirrup was subjected to a cyclic load varying between −6000 N (compression) and 6000 N (tension). The specimen underwent compressive loading over the entire displacement range before entering the tensile phase, with this loading sequence alternating continuously. As shown in Figure 13, hysteresis curves of both numerical and experimental techniques are displayed. Numerical and experimental tests agreed in terms of crack size and path which shows good reproducibility. Nevertheless, a slight discrepancy between the two tests was noted, likely due to experimental uncertainties inherent in the physical testing and the reliance on numerical parameters obtained from the literature for the simulation.

The experimental results presented in this study demonstrate that crack initiation occurs after a relatively low number of cycles under the applied loading conditions, which were designed to simulate accelerated fatigue scenarios relevant to scaffolding applications. While these conditions provide a controlled environment for isolating key failure mechanisms, we acknowledge that further justification is required to align them with real-world service conditions, including variable loading profiles and environmental influences. The micro-hardness measurements, though preliminary, indicate localized hardening near the crack tip, a phenomenon that will be explored in greater depth in future work through detailed microstructural analysis (SEM/optical microscopy) and statistical evaluation of measurement variability. Numerically, the XFEM model effectively captures crack initiation and propagation trends, with quantitative validation now provided through direct comparisons of stress distributions, crack growth rates, and cycle counts between experimental and simulated results. Discrepancies observed—such as variations in peak stress and cycle endurance—highlight the need for further refinement, particularly in incorporating real-world complexities like material inhomogeneities and environmental degradation. This study thus establishes a foundational understanding of fatigue behavior in S235JR steel stirrups, while identifying critical avenues for future research to enhance predictive accuracy and practical applicability. While the current study provides key insights into the fatigue behavior of S235JR steel stirrups—highlighting crack initiation at 286 cycles (without pin) and ultimate failure at 660 cycles (with pin)—it is important to acknowledge the limitations imposed by the small sample size. The results presented are based on representative tests and, as such, may not fully account for the statistical variability inherent in fatigue performance due to material inhomogeneities, manufacturing inconsistencies, or environmental factors. Future work will address this by expanding the experimental dataset to include a larger number of samples and a broader range of loading conditions, thereby enhancing the reliability and generalizability of the findings. This will provide a more comprehensive understanding of the fatigue life distribution and failure mechanisms in scaffolding applications.

## 4. Conclusions

The present study conducted a detailed evaluation of the fatigue behavior of S235JR steel scaffolding stirrups, combining experimental testing and numerical simulations using the Extended Finite Element Method (XFEM) in ABAQUS. Through this approach, we identified and quantified the critical stress concentration zones under compression–tension loading, revealing that the maximum force magnitude reached 6000 N, while the stirrup endured an average of 660 cycles before the onset of macroscopic damage. The Johnson–Cook model was employed to compare experimental and numerical hysteresis behavior, demonstrating a strong correlation in stress distribution patterns, though discrepancies in peak stress values and cycle counts were observed, likely due to microstructural variations and experimental constraints. Crack growth was meticulously characterized across all stages, with a particular focus on the initiation phase at stress concentration sites and the propagation phase governed by cyclic plastic deformation. These findings provide a quantitative foundation for understanding the fatigue life of scaffolding components under controlled laboratory conditions.

While this study offers critical insights into the localized fatigue behavior of the stirrups, it is important to emphasize that the conclusions are specific to the tested material and loading conditions. Broader claims regarding engineering service life improvement or general safety assessments would require additional experimental validation, including environmental effects, variable loading scenarios, and long-term field testing. Future work will expand on these findings by integrating microstructural analysis (SEM/optical microscopy) and refining the XFEM model to incorporate environmental degradation mechanisms. The current results underscore the necessity for proactive fatigue monitoring in scaffolding systems and highlight the potential of numerical models to predict and mitigate failure risks in structural components. However, the scope of these conclusions remains limited to the parameters and conditions evaluated herein.

## Figures and Tables

**Table 1:** Chemical composition of S235JR steel.

Chemical Composites	S234 JR Steel
C	0.040
Si	0.018
Mn	0.232
P	0.015
S	0.017
Cr	0.098
Ni	0.111
V	0.001
Al	0.031
Cu	0.409
Nb	0.001
Ti	0.001
Fe	balance

## Data Availability

The original contributions presented in this study are included in the article. Further inquiries can be directed to the corresponding author.
